# Redistribution of Monocarboxylate 1 and 4 in Hippocampus and Spatial Memory Impairment Induced by Long-term Ketamine Administration

**DOI:** 10.3389/fnbeh.2020.00060

**Published:** 2020-04-17

**Authors:** Runtao Ding, Yaqing Tan, Ao Du, Gehua Wen, Xinghua Ren, Hui Yao, Weishu Ren, Huairu Liu, Xiaolong Wang, Hao Yu, Jun Yao, Baoman Li, Guohua Zhang, Yan Lu, Xu Wu

**Affiliations:** ^1^School of Forensic Medicine, China Medical University, Shenyang, China; ^2^Department of Forensic and Medical Laboratory, Jining Medical University, Jining, China; ^3^Key Laboratory of Health Ministry in Congenital Malformation, The Affiliated Shengjing Hospital of China Medical University, Shenyang, China

**Keywords:** monocarboxylate transporters, astrocyte-neuron lactate shuttle, cognitive dysfunction, ketamine, long-term administration, hippocampal

## Abstract

The monocarboxylate transporters (MCTs) MCT1, MCT2, and MCT4 are essential components of the astrocyte-neuron lactate shuttle (ANLS), which is a fundamental element of brain energetics. Decreased expression of MCTs can induce cognitive dysfunction of the brain. In the present study, we established a mouse model of long-term ketamine administration by subjecting mice to a 6-month course of a daily intraperitoneal injection of ketamine. These mice demonstrated learning and memory deficits and a significant decline in MCT1 and MCT4 proteins in the hippocampal membrane fraction, while cytoplasmic MCT1 and MCT4 protein levels were significantly increased. In contrast, the levels of global MCT2 protein were significantly increased. Analysis of mRNA levels found no changes in MCT1/4 transcripts, although the expression of MCT2 mRNA was significantly increased. We suggest that redistribution of hippocampal MCT1 and MCT4, but not MCT2 up-regulation, may be related to learning and memory deficits induced by long-term ketamine administration.

## Introduction

The monocarboxylate transporters (MCTs) family is composed of several members that mediate fluxes of metabolites, such as L-lactate, pyruvate and ketone bodies (Halestrap and Price, 1999). It is generally accepted that rodent astrocytes mainly express MCT1 and MCT4, whereas neurons mainly express MCT2 (Debernardi et al., 2003; Khatri and Man, 2013). The activity of the astrocyte-neuron lactate shuttle (ANLS) is involved in brain energetic support, and the process of lactate transport from astrocytes to neurons, which are important in neuronal energy support, depends on the activity of MCTs (Whitlock et al., 2006; Herrero-Mendez et al., 2009). Abnormal expression of MCTs, especially MCT1 and MCT4, can lead to a series of central nervous system (CNS) dysfunctions. Transgenic mice with reduced MCT1 expressions exhibited motoneuron toxicity (Lee et al., 2012). Meanwhile, an *in vitro* study demonstrated that transfected astrocytes that overexpressed MCT4 would significantly hamper the growth ability of primary neurons, when co-culturing with each other (Hong et al., 2019). On the other hand, MCTs expression disorder is also observed in a lot of brain dysfunctions (Leroy et al., 2011; Lauritzen et al., 2012), together with delayed energic support dysfunction (Loane and Faden, 2010).

Ketamine is a non-competitive, N-methyl-D-aspartate (NMDA) receptor antagonist, which is a popular general anesthetic with anti-depressive properties. In recent decades, however, ketamine has increasingly become an addictive recreational drug (Sassano-Higgins et al., 2016; Wang et al., 2019). To date, few epidemiological studies have focused on cognitive impairment induced by long-term ketamine administration. It is reported that ketamine abuse leads to psychiatric disorders and cognitive dysfunction such as working memory or episodic memory impairments (Morgan et al., 2010; Carter et al., 2013; de Souza et al., 2019). In rodent models, aberrant neuronal apoptosis has been observed in the developing CNS after acute ketamine treatment (Ikonomidou et al., 1999; Wang et al., 2014; Obradovic et al., 2018), yet the underlying mechanisms remain to be elucidated. Recent studies reported that short-term ketamine abuse disturbs cognitive function by acting through alpha-amino-3-hydroxy-5-methyl-4-isoxazolepropionic acid (AMPA) and NMDA receptors (Xu and Lipsky, 2015; Ranganathan et al., 2017; Hasegawa et al., 2019). Our previous study similarly demonstrated that chronic exposure to ketamine initiated the abnormal expression of AMPA and NMDA receptors in the injured hippocampus of mice (Ding et al., 2016).

Excitatory glutamatergic neurotransmission requires energetic support, which in part, is provided through the ANLS; thus is critically involved in synaptic plasticity, learning, and memory (Bergersen et al., 2005; Belanger et al., 2011; Khatri and Man, 2013). Abnormal expression of MCT can lead to cognitive dysfunction and learning and memory impairment (Pérez-Escuredo et al., 2016). Learning and memory deficits were found in rats when hippocampal MCT1 and MCT4 expression was inhibited (Suzuki et al., 2011; Sun et al., 2018). In rat models of Alzheimer’s disease (AD), hippocampal MCT2 and lactate content was significantly decreased after bilateral hippocampal Aβ_25–35_ injection, and accompanied by learning and memory deficits (Lu et al., 2015). Accumulation of hyperphosphorylated tau (a pathological marker of AD) have been identified in the cerebral cortex after long-term ketamine administration (Zheng et al., 2019).

Thus, long-term ketamine administration might be associated with an abnormality in cerebral MCTs leading to dysfunction of the hippocampus. However, little is known about how exposure to ketamine affects the expression of MCTs. In this study, we utilize a mouse model of long-term ketamine administration to examine learning and memory deficits and the changes of MCT1, MCT4, and MCT2 expression levels in the membrane and cytoplasmic fractions of the hippocampus.

## Materials and Methods

### Animals and the Model of Long-Term Ketamine Administration

**Figure 1:**
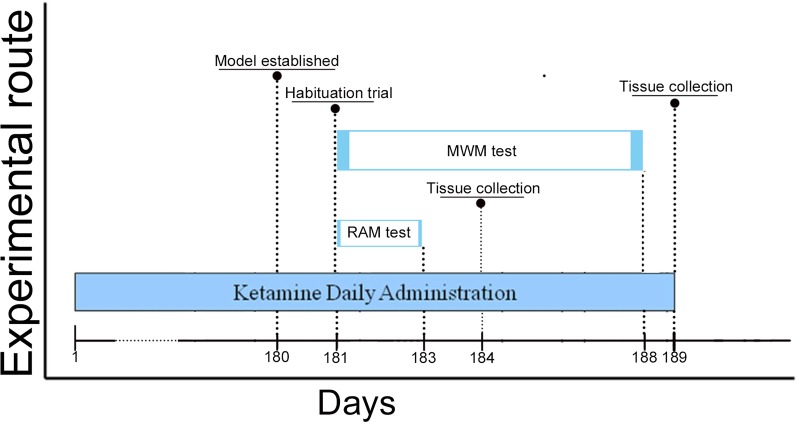
Schedule of ketamine administration and behavioral assessment. Ketamine daily administration for 180 days (6 months). Morris Water Maze (MWM) test began on Day 181 and lasted until Day 188 of the study, the Radial Arm Maze (RAM) test started on Day 181 and lasted until on Day 183. We surveyed the sessions of escape latency (Day 183–187) and probe trials (Day 188) of mice to assess for spatial memory performance in the MWM test. Meanwhile, total errors and time spent to finish a session (Day 92–93 and Day 182–183) of mice in the RAM test were conducted to assess for spatial working memory performance. Animals were euthanized on Day 184 and 189, and brain tissue was collected in the −80°C freezer.

Ninety male C57BL/6J mice (2 months old) from the Laboratory Animal Centre of China Medical University (license number: SCXK (Liao) 2018–0004), weight 17–22 g, were housed 3–4 per cage and maintained on a 12 h light/dark cycle (lights out at 6:00 PM). The mice had unlimited access to water and food in their home cages. The animal experiments were approved by the Animal Research Ethics Committee of China Medical University following China’s experimental animal administrative regulations and conducted in the Laboratory Animal Centre of China Medical University [license number: SYXK (Liao) 2018-0008]. All efforts were made to minimize the number of animals used and to reduce their suffering. The entire experimental paradigm was carried out according to the schedule shown in Figure 1.

The establishment of a long-term ketamine administration model is described as our previous articles (Ding et al., 2016). Briefly, mice received daily intraperitoneal injection of ketamine (ketamine hydrochloride, Fujian Gutian Pharmaceutical Company Limited, Gutian, Fujian, People’s Republic of China, dissolved in physiological saline) or equal volumes of physiological saline on 07:00 am in the light cycle for 180 consecutive days. Three groups of mice were randomly assigned, with 30 mice per group: a **saline** group that was injected with physiological saline; a **ket1** group that was injected with 30 mg/kg of ketamine; and a **ket2** group that was injected with 60 mg/kg of ketamine. We diluted the original ketamine hydrochloride into the 30 mg/kg and 60 mg/kg doses of ketamine according to the mouse’s weight (the volume was recorded); the details are given in the Supplementary Table S1. Mice were weighed every Monday and Thursday. The average weights of the three groups were 23.1 g (**saline** group), 24.6 g (**ket1** group), and 23.8 g (**ket2** group) on the last day of the model establishment. There is no significant difference in changes in weight in the three groups (Supplementary Figure S1). During the experiment, eight mice died: two in the **saline** group; three in the **ket1** group; and three in the **ket2** group. The causes of death were examined post-mortem: four had bleeding in the abdominal cavity; two had visceral organ rupture (two hepatic ruptures and one kidney rupture); and two showed no apparent signs of injury or disease.

### Behavioral Tasks

#### Morris Water Maze (MWM) Test and Radial Arm Maze (RAM) Test

Twenty-four hours after the 180-day ketamine administration procedure (in the day 181), behavioral tests are carried out 3 h after ketamine administration. Ketamine administration continued throughout the whole behavioral tests. The MWM test and RAM test were both carried out in the day 181, 42 mice (**saline** group, *n* = 14; **ket1** group, *n* = 14; **ket2** group, *n* = 14) were used in MWM test and the other 40 mice (**saline** group, *n* = 14; **ket1** group, *n* = 13; **ket2** group, *n* = 13) were used in RAM test. and the entire process was monitored using the SMART™ tracking software program (San Diego Instruments, San Diego, CA, USA). All the mice exhibited a normal state before and during the behavioral tests, which were performed in a quiet room. Any mouse that exhibited passivity or thigmotaxic tracks was excluded from the analysis.

The MWM test was conducted as described previously, with slight modification (Ding et al., 2016). Briefly, a visible platform was used to exclude mice that might have visual or locomotive impairments, and to enable the mice to habituate to the circular apparatus (100 cm diameter). Mice were trained for two consecutive days (four trials per day). The walls of the maze were posted with spatial cues of different colors and shapes. A white plastic platform (8 cm diameter) protruded 2 cm above the surface of the water. The mice swam freely for 60 s, and were permitted to stay on the platform for 20 s after landing. Mice that were unable to find the visible platform were placed on the platform for an extra 10 s. Most of the mice exhibited good habituation and mobility.

Subsequently, each mouse was given three trials per day for five consecutive days in a pool with a hidden platform that was 0.8 cm below the surface of the opaque water. The location of the platform was fixed, but the starting point changed on every trial to avoid track memorization. Whether the mice successfully found the platform within 60 s or failed to do so in 60 s, they were allowed to rest on the platform for 10 s. The latency to the target (escape latency) was recorded.

Each mouse was given a memory retention test 24 h after the last training trial with the hidden platform. The platform was removed for the memory test and the mice were allowed to swim freely for 60 s. The number of times the mouse swam over the previous platform location was counted.

The RAM test was performed following the previous tests (Ding et al., 2016). Briefly, food was withheld for 24 h. During each session, the mice could freely visit the arms of the maze to obtain food pellets until the eight arms had been visited or 20 min run out. The locomotion of the animals was monitored *via* the SMART™ tracking software program. The numbers of non-visited arms and re-entry into arms were scored as working memory errors and summed. The apparatus was cleaned with 25% ethanol solution after each trial.

### Hippocampal Membrane Protein Fractionation and Western blot

Hippocampi were isolated, membrane and cytoplasmic proteins were extracted by ProteoExtract Transmembrane Protein Extraction Kit (71772, Millipore, Billerica, MA, USA) and protease/phosphatase inhibitors (Sigma–Aldrich, St. Louis, MO, USA). Then 20 μg of each lysate was separated on SDS-PAGE gels and transferred to PVDF membranes (Bio-Rad, Hercules, CA, USA). After 5% BSA blocking, the blots were incubated with primary antibodies and appropriate secondary antibodies, and visualized with a chemiluminescence system (Tanon 5200, Shanghai, China). Data analyses of blots were performed by using GraphPad Prism 6.01 software. The following antibodies were used: anti-MCT1 (1:400, sc-365501, Santa Cruz Biotechnology, Santa Cruz, CA, USA); anti-MCT2 (1:300, sc-271093, Santa Cruz Biotechnology, Santa Cruz, CA, USA); anti- MCT4 (1:300, sc-376140, Santa Cruz Biotechnology, Santa Cruz, CA, USA); anti-GAPDH (1:5,000, ab181602, Abcam).

### Total RNA Extraction and Quantitative Real-Time PCR

Total RNA was isolated from the hippocampi with RNAiso Plus (9108, Takara Biotechnology, Shiga, Japan) according to the manufacturer’s directions. All RNA samples were under the check of OD values A260/A280 ranged from 1.8 to 2.0. Total RNA was reverse-transcribed into cDNA utilizing the PrimeScript™ RT reagent Kit (RR037A, Takara Biotechnology, Shiga, Japan). After cDNA amplification (20-μl reaction mixture), quantitative real-time PCR (qPCR) with the sequence-specific primer pairs for MCT1, MCT2, MCT4, and Gapdh was performed by Applied Biosystems 7500 Real-Time PCR System using SYBR^®^ PrimeScript™ RT-PCR Kit (RR081A, Takara Biotechnology, Shiga, Japan). Negative controls (ddH2O instead of cDNA) were also added to exclude potential contaminations during each run. The qPCR procedure was repeated at least three times for each sample. The detailed information of the primer sequences was shown in the Supplementary Table S2.

### Statistical Analysis

Data were expressed as mean ± SEM and analyzed by PRISM 6.01 software. All data were tested for normal distribution by the Shapiro-Wilk test (*p* < 0.05) and they were equal variance. One-way ANOVA, repeated measures Two-way ANOVA is used for data statistics. *Post hoc* comparisons were performed if there is a significant difference among means. The data statistic of latency time in probe trials of the MWM test is analyzed by repeated measures two-way ANOVA. The other data analysis uses One-way ANOVA. Difference associated with *p < 0.05, **p < 0.01 or ^#^*p* < 0.05, ^##^*p* < 0.01 is considered as statistically significant.

## Results

### Acute Performance of Mice After Daily Ketamine Injection

The mice treated with the ketamine at 60 mg/kg exhibited a transient dystaxia state (starting from 2 min after ketamine administration and the whole dystaxia state lasted nearly 5 min), including drunken gait, slipping, limbs tremor and disturbances in balance. whereas those treated with 30 mg/kg exhibited hyperactivity (see short videos in the Supplementary Video S1).

### Six Months of Ketamine Administration Impairs Learning and Memory

**Figure 2:**
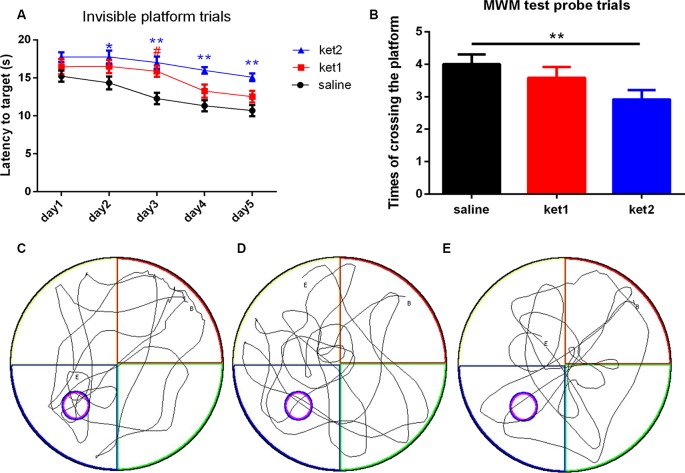
Spatial memory performance of mice in the MWM test following 6 months of ketamine administration with different doses of ketamine 30 mg/kg and 60 mg/kg. **(A)** Repeated measures 2-way ANOVA was used in the analysis of latency time in probe trials. Main effect of treatment (saline, Ket1, Ket2), time)day1-day5) and interactions of treatment × time are reported as followed: treatment *F*_(4,52)_ = 6.32, **p < 0.01; time *F*_(2,26)_ = 6.919, **p < 0.01; interactions of treatment × time *F*_(8,104)_ = 0.758, *p* = 0.8475. Significant increase of latency time to reach the target in MWM test invisible platform trails in ket2 group in the training day 2, 3, 4, 5 after 6 months of administration paradigm (*p < 0.05, **p < 0.01) and ket1 group saw an increase of latency time to reach the target only in the training day 3 (^#^*p* < 0.05). **(B)** A significant decline of the times of crossing the former platform location was observed in the ket2 group comparing with the control group (*F*_(2,39)_ = 5.474, **p < 0.01). **(C–E)** Representative swimming paths by mice with different treatments in probe trial tests (saline *n* = 14, ket1 *n* = 14, ket2 *n* = 14).

A significant increase in latency to target in the MWM test was seen in the **ket2** group on days 2, 3, 4, and 5 after the 6-month treatment, compared with the **saline** group (Figure 2A; *p < 0.05, **p < 0.01, repeated-measures 2-way ANOVA), and a significant decrease in the numbers of platform crossings was observed in the probe trials in the **ket2** group, compared with the **saline** group (Figure 2B; *p < 0.05, One-way ANOVA). Although the **ket1** group exhibited an increase in latency to target on day 3, there was no statistically significant difference between the **ket1** group and the **saline** group on the rest of the trials (Figures 2A,B). Behavioral tracking consistently revealed that the mice of the **ket2** group displayed less exploration in the former target location and the target quadrant than the mice of the **saline** group (Figures 2C–E).

**Figure 3:**
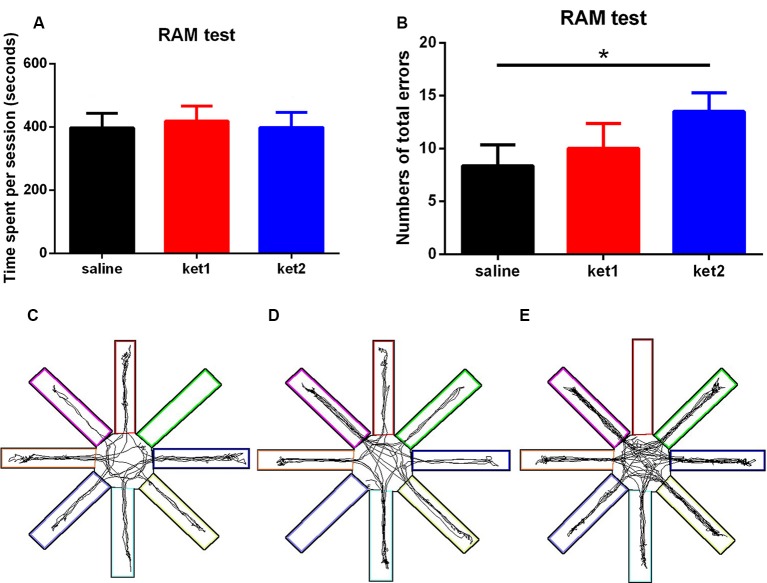
Spatial working memory performance of mice in RAM tests following 6 months of ketamine administration with different doses of ketamine 30 mg/kg and 60 mg/kg. **(A)** No significance of time spent in finishing a session was seen in three groups. **(B)** Significant increase of total errors in one session to accomplish a RAM test in ket2 group comparing with the control group (*F*_(2,37)_ = 3.718, *p < 0.05). **(C–E)** Representative searching tracks of mice with different treatments in the RAM test acquisition trails (saline *n* = 14, ket1 *n* = 13, ket2 *n* = 13).

The RAM test demonstrated that the **ket2** group had a significant increase in total errors compared to the **saline** group (Figure 3B, *p < 0.05, One-way ANOVA). However, the time it took to finish a session was not significantly different among the three groups (Figure 3A), thus, excluding the possibility that locomotion deficits interfered with spatial working memory. Figures 3C–E illustrate the tracks of the mice in the three groups during the RAM test.

### Plasmalemmal Expression of MCT1, MCT2, and MCT3 After 6-Month Administration of Ketamine

**Figure 4:**
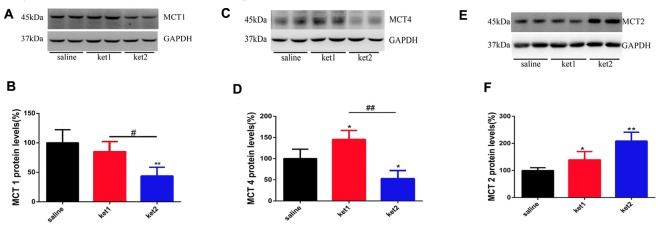
Changes of hippocampal membrane monocarboxylate transporters 1 (MCT1), MCT4 and MCT2 protein levels after 6 months of ketamine administration in different groups as revealed by Western blot. **(A,B)** Significant decline of MCT1 expression levels were seen in ket2 group comparing with ket1 group and control group respectively (*F*_(2,27)_ = 3.405, ^#^*p* < 0.05 and *F*_(2,27)_ = 5.823, **p < 0.01, respectively). **(C,D)** Significant decline of MCT4 expression levels were seen in ket2 group comparing with control group and ket1 group respectively (*F*_(2,27)_ = 3.442, *p < 0.05 and *F*_(2,27)_ = 5.604, ^##^*p* < 0.01, respectively). Obviously increase of MCT4 expression levels were seen in ket1 group comparing with control group (*F*_(2,27)_ = 3.482, *p < 0.05). **(E,F)** Significant increase of MCT2 expression levels were seen in ket2 and ket1 group comparing with control group respectively (*F*_(2,27)_ = 6.104, **p < 0.01; *F*_(2,27)_ = 3.707, *p < 0.05, respectively; saline *n* = 10, ket1 *n* = 10, ket2 *n* = 10).

The levels of MCT1 protein in hippocampal membrane fractions were significantly decreased in the **ket2** group compared to the **saline** and **ket1** groups (Figure 4B, **p < 0.01, ^#^*p* < 0.05, respectively, One-way ANOVA). The expression of MCT2 protein was increased in both the **ket1** and **ket2** mice compared with the **saline** mice (Figure 4F, *p < 0.05, **p < 0.01, respectively, One-way ANOVA), whereas MCT4 protein levels were decreased in the **ket2** mice compared with **saline** and **ket1** mice (Figure 4D, *p < 0.05, ^##^*p* < 0.01, respectively, One-way ANOVA). In contrast, the MCT4 protein level was increased in the **ket1** mice compared to the **saline** mice (Figure 4D, *p < 0.05, One-way ANOVA).

### Cytosolic Expression of MCT1, MCT2, and MCT4 After Long-Term Ketamine Administration

**Figure 5:**
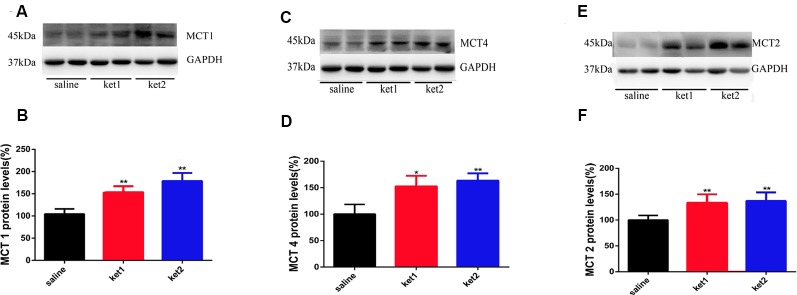
Changes of hippocampal cytoplasm MCT1, MCT4 and MCT2 protein levels after 6 months of ketamine administration in different groups as revealed by Western blot. **(A,B)** Significant increase of MCT1 expression levels were seen in ket2 and ket1 group comparing with control group respectively (*F*_(2,27)_ = 5.943, *F*_(2,27)_ = 5.477, **p < 0.01, respectively). **(C,D)** Significant increase of MCT4 expression levels were seen in ket2 and ket1 group comparing with control group (*F*_(2,27)_ = 3.607, *p < 0.05; *F*_(2,27)_ = 5.882, **p < 0.01, respectively). **(E,F)** Significant increase of MCT2 expression levels were seen in ket2 and ket1 group comparing with control group respectively (*F*_(2,27)_ = 5.742, **p < 0.01; saline *n* = 10, ket1 *n* = 10, ket2 *n* = 10).

Cytoplasmic MCT1 protein levels were significantly increased in the **ket1** and **ket2** groups (Figure 5B, **p < 0.01, One-way ANOVA). Similar increases in cytoplasmic expression were found for the MCT2 and MCT4 proteins (Figures 5D,F, **p < 0.01, **p < 0.01, One-way ANOVA).

### mRNA Expression of MCT1, MCT2, and MCT4 After Long-Term Ketamine Administration

**Figure 6:**
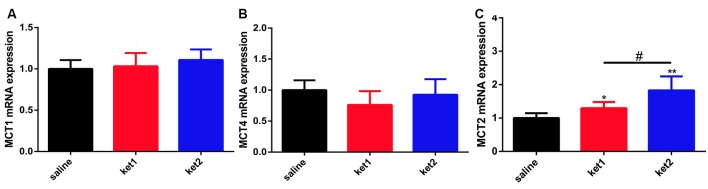
Changes of hippocampal MCT1, MCT4 and MCT2 mRNA levels after 6 months of ketamine administration in different groups. **(A)** No significance of MCT1 mRNA expression levels were seen in three groups. **(B)** Significant increase of MCT2 mRNA expression levels were seen in ket2 and ket1 group comparing with control group (*F*_(2,33)_ = 3.709, *p < 0.05, *F*_(2,33)_ = 3.461, ^#^*p* < 0.05 and *F*_(2,33)_ = 5.758, **p < 0.01, respectively). **(C)** No significance of MCT4 mRNA expression levels were seen in three groups (saline *n* = 12, ket1 *n* = 12, ket2 *n* = 12).

We conducted real-time qPCR tests to determine if mRNA levels had changed in hippocampal MCT1, MCT4, and MCT2 after long-term ketamine administration. The results revealed no significant difference in MCT1 and MCT4 among the 3 groups (Figures 6A,C). However, significant increases in MCT2 mRNA levels were seen in the **ket1** and **ket2** groups compared with the **saline** group (Figure 6B, *p < 0.05, **p < 0.01, One-way ANOVA).

## Discussion

Cerebral MCTs dependent ANLS is indispensable for brain energy supports and important for neuronal plasticity and the process of learning and memory (Bergersen et al., 2005; Belanger et al., 2011; Khatri and Man, 2013). Abnormal MCTs expression is involved in neurodegenerative disorders (Lee et al., 2012; Hong et al., 2019). Ketamine and other addictive drug abuse may lead to neurodegeneration and neurotoxicity (Ikonomidou et al., 1999; Moszczynska and Callan, 2017; Li et al., 2019). Thereby, these researches indicated that changes in cerebral MCTs expressions may be relevant to neurotoxicity induced by ketamine abuse. However, little is known about how the expression of MCTs is affected in exposure to long-term ketamine administration.

Here, the findings of the present study showed that the long-term administration of ketamine induces dose-dependent impairments in learning and memory and alterations in the expression of hippocampal MCTs, which are necessary for cerebral ANLS and cognitive function. Long-term administration of ketamine-induced a decline in MCT1 and MCT4 membrane expression but the increment in cytoplasmic expression. The long-term administration of ketamine-induced hippocampal MCT2 up-regulation by enhancing the transcription of MCT2 mRNA.

Cognitive impairments induced by ketamine abuse have been reported in humans (Carter et al., 2013). Interestingly, low doses of ketamine produce neuroprotective effects and cognitive improvements in patients with major depressive disorder and rodent models (Hasegawa et al., 2019; Zheng et al., 2019), whereas ketamine 80 mg/kg produces learning and memory deficits, compared to 30 mg/kg (Wang et al., 2014). Moreover, mice exposed to a 30 mg/kg dose of ketamine for 6 months do not exhibit learning or memory impairments (Yeung et al., 2010). These studies indicate that sub-anesthetic doses of ketamine induce learning and memory impairment in a dose-dependent manner. Coincidently, our present study showed that the mice model of long-term ketamine administration exhibited a significant dose-dependent impairment in learning and memory at a 60 mg/kg dose of ketamine, but not at a 30 mg/kg dose.

MCTs that take part in ANLS are indispensable for normal CNS energy support, especially for CNS glutamatergic synaptic plasticity, which is crucial for learning and memory; and disturbances in cerebral MCT expression lead to ANLS and cognitive dysfunction (Halestrap and Price, 1999; Belanger et al., 2011; Suzuki et al., 2011; Khatri and Man, 2013). The expression of MCT1 and MCT4 is mainly localized in astrocytes (Pellerin et al., 2005; Solís-Maldonado et al., 2018), and the expression of MCT2 occurs mainly in neurons (Vannucci and Simpson, 2003). Then we determined hippocampal astrocyte-located expression of MCT1 and MCT4 and neuron-located MCT2 expression to check the alterations of hippocampal MCT systems after long-term exposure to ketamine.

Our results showed that the levels of hippocampal membrane MCT1 and MCT4 proteins significantly declined after long-term administration of 60 mg/kg ketamine. However, the levels of the hippocampal membrane MCT2 protein were significantly increased after the same administration paradigm. We assumed that long-term ketamine administration might impair hippocampal ANLS lactate transmission by reducing the expression of membrane MCT1 and MCT4 and ultimately, induce learning and memory deficits in mice. The reduced membrane MCT1 and MCT4 expression would have caused a lactate supply shortage, and membrane MCT2 expression may have up-regulated in response.

We further checked the hippocampal cytoplasmic protein and global mRNA levels of MCTs and found that the levels of hippocampal cytoplasmic MCT1 and MCT4 proteins significantly increased, but no significant change was found in the levels of hippocampal global MCT1 or MCT4 mRNA in real-time qPCR tests after long-term ketamine administration. The results of the hippocampal cytomembrane and cytoplasm MCT1 and MCT4 protein levels and global mRNA levels imply that the long-term administration of ketamine at 60 mg/kg induced subcellular MCT1 and MCT4 redistribution between the membrane and the cytoplasm. Also, the hippocampal cytoplasm protein expression of MCT2 was significantly increased, and the hippocampal global MCT2 mRNA level significantly increased after the administration of ketamine at 30 mg/kg and 60 mg/kg. These results indicate that the long-term administration of ketamine promoted the global expression of hippocampal MCT2 protein levels, together with an increase in membrane expression.

MCT1, 2 and 4 are widely expressed in a variety of organs, including the brain, heart, muscle and so forth (Halestrap and Wilson, 2012). Alterations of peripheral MCTs expression could induce a diversity of locomotive dysfunctions. MCT1 deficiency in muscle is reported to be associated with muscle cramping after strenuous exercise, along with a defect in lactate efflux from muscle (Aoi and Marunaka, 2014). Suppression or dysfunction of MCT4 may restrict lactic acid efflux from skeletal muscle, following with the decline of muscle pH, which may lead to impairment of muscle function (Juel and Halestrap, 1999; Bonen, 2001; Halestrap and Meredith, 2004). Yet the present data of cognitive tasks demonstrated that mice exhibited no significant difference in locomotive activities after long-term ketamine administration, which implied that the changes of peripheral MCTs expression on physical ability are limited on the performance of behavioral tests.

Abnormal expression of MCTs in CNS may affect both limbic and hippocampal related cognitive functions. MCTs are reported to be indispensable for amygdala-dependent fear memory acquisition, as MCT inhibitor 4-CIN could significantly reduce the freezing values during contextual fear conditioning training (Kong et al., 2017); meanwhile, MCT1 and MCT2 is indispensable for rewarding memory of cocaine relapse (Zhang et al., 2016). However, the results of the current behavioral tasks showed that mice exhibited spatial memory impairments, which is closely related to functions of the hippocampus other than limbic system-dependent cognitive functions that are predominantly relevant to emotion, addiction and reward. Although there is no direct published evidence demonstrate that complex of the abnormal MCTs expression in the hippocampus and other parts of limbic system may induce the present spatial memory deficits, it is reported that increased MCT2 but reduced MCT4 levels were found in posterior cingulate (one part of the limbic system) of young adult APOE4 carriers, those who have a huge genetic risk for late-onset AD (Perkins et al., 2016). Meanwhile, research on AD rat models has found decreased levels of expression of hippocampal MCT2 and obvious cognitive impairments, which indicates that an impairment in the ANLS downstream neuronal MCT2 energy acquisition process might be involved in AD-related learning and memory deficits (Lu et al., 2015). These evidences implied that abnormal MCTs expression in the hippocampus together with other parts of the limbic system may simultaneously play roles in cognitive deficits. However, a mice model of Huntington’s disease has found an increase in lactate up-take and the catalytic efficiency of hippocampal membrane MCT2, while no significant increases have been found in protein or mRNA levels (Solís-Maldonado et al., 2018).

Interestingly, although the mice in our study exhibited an increase in MCT4 and MCT2 protein levels and MCT2 mRNA after the long-term administration of 30 mg/kg of ketamine, the mice did not show obvious learning or memory improvements. ICR mice administered 30 mg/kg of ketamine for 3 months have been found to exhibit increased dopamine levels (Tan et al., 2011), whereas C57 mice given the same dose of ketamine for 6 months have been found to show no cognitive impairment (Sun et al., 2011), even though the mice showed hyper-phosphorylation of tau and apoptosis in the prefrontal and entorhinal cortex (Yeung et al., 2010). These results indicate that: on one hand, a dose of 30 mg/kg of ketamine is not sufficient to induce learning and memory deficits in mice models; and, on the other hand, a dose of 30 mg/kg of ketamine is probably an intermediate dose that mediates complicated and “contradictory” biochemical and physiological activities. As acute treatment with a lower dose of ketamine (10 mg/kg) can reverse depressive-like behaviors in mice by up-regulating hippocampal AMPA receptor expression (Tosta et al., 2019), While a higher dose of ketamine (100 mg/kg) induces neurotoxicity (Chatterjee et al., 2012).

In summary, our present study primarily demonstrated that the long-term administration of ketamine can lead to dose-dependent learning and memory deficits and alterations in hippocampal MCTs, including an ANLS upstream, decrease in membrane MCT1 and MCT4 and increase in downstream MCT2. We also found that the long-term administration of ketamine-induced abnormal distributions of subcellular hippocampal MCT1 and MCT4 by removing them from the membrane to cell plasma, but enhanced the global expression of MCT2. We suspect that it is the reduced levels of hippocampal membrane MCT1 and MCT4 that are involved in the cognitive deficits observed in the long-term ketamine administration. However, the potential mechanism of MCT1 and MCT4 alterations and ANLS dysfunction in long-term, ketamine-induced learning and memory deficits remains to be elucidated.

## Data Availability Statement

The datasets generated for this study are available on request to the corresponding author.

## Ethics Statement

The animal study was reviewed and approved by the Animal Research Ethics Committee of China Medical University.

## Author Contributions

YL and XWu designed the experiments. RD and YT analyzed the data and drafted the manuscript. RD, AD, GW, XR, and HYa performed the behavior tests, YT, WR, and HL supported their works in protein and mRNA experiments. XWa, HYu, and JY did the work of brain tissue preparation. BL, GZ, YL, and XWu revised the manuscript. All authors read and approved the final manuscript.

## Conflict of Interest

The authors declare that the research was conducted in the absence of any commercial or financial relationships that could be construed as a potential conflict of interest.

## References

[B1] Aoi W. Marunaka Y. (2014). Importance of pH homeostasis in metabolic health and diseases: crucial role of membrane proton transport. Biomed. Res. Int. 2014:598986. 10.1155/2014/598986 25302301 PMC4180894

[B2] Belanger M. Allaman I. Magistretti P. J. (2011). Brain energy metabolism: focus on astrocyte-neuron metabolic cooperation. Cell Metab. 14, 724–738. 10.1016/j.cmet.2011.08.016 22152301

[B3] Bergersen L. H. Magistretti P. J. Pellerin L. (2005). Selective postsynaptic co-localization of MCT2 with AMPA receptor GluR2/3 subunits at excitatory synapses exhibiting AMPA receptor trafficking. Cereb. Cortex 15, 361–370. 10.1093/cercor/bhh138 15749979

[B4] Bonen A. (2001). The expression of lactate transporters (MCT1 and MCT4) in heart and muscle. Eur. J. Appl. Physiol. 86, 6–11. 10.1007/s004210100516 11820324

[B5] Carter L. P. Kleykamp B. A. Griffiths R. R. Mintzer M. Z. (2013). Cognitive effects of intramuscular ketamine and oral triazolam in healthy volunteers. Psychopharmacology 226, 53–63. 10.1007/s00213-012-2883-x 23096769 PMC3572303

[B6] Chatterjee M. Verma R. Ganguly S. Palit G. (2012). Neurochemical and molecular characterization of ketamine-induced experimental psychosis model in mice. Neuropharmacology 63, 1161–1171. 10.1016/j.neuropharm.2012.05.041 22683513

[B7] de Souza I. Meurer Y. Tavares P. M. Pugliane K. C. Lima R. H. Silva R. H.. (2019). Episodic-like memory impairment induced by sub-anaesthetic doses of ketamine. Behav. Brain Res. 359, 165–171. 10.1016/j.bbr.2018.10.031 30359643

[B8] Debernardi R. Pierre K. Lengacher S. Magistretti P. J. Pellerin L. (2003). Cell-specific expression pattern of monocarboxylate transporters in astrocytes and neurons observed in different mouse brain cortical cell cultures. J. Neurosci. Res. 73, 141–155. 10.1002/jnr.10660 12836157

[B9] Ding R. Li Y. Du A. Yu H. He B. Shen R.. (2016). Changes in hippocampal AMPA receptors and cognitive impairments in chronic ketamine addiction models: another understanding of ketamine CNS toxicity. Sci. Rep. 6:38771. 10.1038/srep38771 27934938 PMC5146946

[B10] Halestrap A. P. Meredith D. (2004). The SLC16 gene family-from monocarboxylate transporters (MCTs) to aromatic amino acid transporters and beyond. Pflugers Arch. 447, 619–628. 10.1007/s00424-003-1067-2 12739169

[B11] Halestrap A. P. Price N. T. (1999). The proton-linked monocarboxylate transporter (MCT) family: structure, function and regulation. Biochem. J. 343, 281–299. 10.1042/bj3430281 10510291 PMC1220552

[B12] Halestrap A. P. Wilson M. C. (2012). The monocarboxylate transporter family—role and regulation. IUBMB Life 64, 109–119. 10.1002/iub.572 22162139

[B13] Hasegawa S. Yoshimi A. Mouri A. Uchida Y. Hida H. Mishina M.. (2019). Acute administration of ketamine attenuates the impairment of social behaviors induced by social defeat stress exposure as juveniles *via* activation of α-amino-3-hydroxy-5-methyl-4-isoxazolepropionic acid (AMPA) receptors. Neuropharmacology 148, 107–116. 10.1016/j.neuropharm.2018.12.020 30590060

[B14] Herrero-Mendez A. Almeida A. Fernández E. Maestre C. Moncada S. Bolaños J. P. (2009). The bioenergetic and antioxidant status of neurons is controlled by continuous degradation of a key glycolytic enzyme by APC/C-Cdh1. Nat. Cell Biol. 11, 747–752. 10.1038/ncb1881 19448625

[B15] Hong P. Zhang X. Gao S. Wang P. (2019). Role of monocarboxylate transporter 4 in Alzheimer disease. Neurotoxicology 76, 191–199. 10.1016/j.neuro.2019.11.006 31738978

[B16] Ikonomidou C. Bosch F. Miksa M. Bittigau P. Vöckler J. Dikranian K.. (1999). Blockade of NMDA receptors and apoptotic neurodegeneration in the developing brain. Science 283, 70–74. 10.1126/science.283.5398.70 9872743

[B17] Juel C. Halestrap A. P. (1999). Lactate transport in skeletal muscle—role and regulation of the monocarboxylate transporter. J. Physiol. 517, 633–642. 10.1111/j.1469-7793.1999.0633s.x 10358105 PMC2269375

[B18] Khatri N. Man H. Y. (2013). Synaptic activity and bioenergy homeostasis: implications in brain trauma and neurodegenerative diseases. Front. Neurol. 4:199. 10.3389/fneur.2013.00199 24376435 PMC3858785

[B19] Kong L. Zhao Y. Zhou W. J. Yu H. Teng S. W. Guo Q.. (2017). Direct neuronal glucose uptake is required for contextual fear acquisition in the dorsal hippocampus. Front. Mol. Neurosci. 10:388. 10.3389/fnmol.2017.00388 29209168 PMC5702440

[B20] Lauritzen F. Perez E. L. Melillo E. R. Roh J. M. Zaveri H. P. Lee T. S.. (2012). Altered expression of brain monocarboxylate transporter 1 in models of temporal lobe epilepsy. Neurobiol. Dis. 45, 165–176. 10.1016/j.nbd.2011.08.001 21856423 PMC3351090

[B21] Lee Y. Morrison B. M. Li Y. Lengacher S. Farah M. H. Hoffman P. N.. (2012). Oligodendroglia metabolically support axons and contribute to neurodegeneration. Nature 487, 443–448. 10.1038/nature11314 22801498 PMC3408792

[B22] Leroy C. Pierre K. Simpson I. A. Pellerin L. Vannucci S. J. Nehlig A. (2011). Temporal changes in mRNA expression of the brain nutrient transporters in the lithium-pilocarpine model of epilepsy in the immature and adult rat. Neurobiol. Dis. 43, 588–597. 10.1016/j.nbd.2011.05.007 21624469 PMC3726264

[B23] Li Y. Ding R. Ren X. Wen G. Dong Z. Yao H.. (2019). Long-term ketamine administration causes Tau protein phosphorylation and Tau protein-dependent AMPA receptor reduction in the hippocampus of mice. Toxicol. Lett. 315, 107–115. 10.1016/j.toxlet.2019.08.023 31470060

[B24] Loane D. J. Faden A. I. (2010). Neuroprotection for traumatic brain injury: translational challenges and emerging therapeutic strategies. Trends Pharmacol. Sci. 31, 596–604. 10.1016/j.tips.2010.09.005 21035878 PMC2999630

[B25] Lu W. Huang J. Sun S. Huang S. Gan S. Xu J.. (2015). Changes in lactate content and monocarboxylate transporter 2 expression in Aβ_25–35_-treated rat model of Alzheimer’s disease. Neurol. Sci. 36, 871–876. 10.1007/s10072-015-2087-3 25647291

[B26] Morgan C. J. Muetzelfeldt L. Curran H. V. (2010). Consequences of chronic ketamine self-administration upon neurocognitive function and psychological wellbeing: a 1-year longitudinal study. Addiction 105, 121–133. 10.1111/j.1360-0443.2009.02761.x 19919593

[B27] Moszczynska A. Callan S. P. (2017). Molecular, behavioral, and physiological consequences of methamphetamine neurotoxicity: implications for treatment. J. Pharmacol. Exp. Ther. 362, 474–488. 10.1124/jpet.116.238501 28630283 PMC11047030

[B28] Obradovic A. L. Atluri N. Dalla Massara L. Oklopcic A. Todorovic N. S. Katta G.. (2018). Early exposure to ketamine impairs axonal pruning in developing mouse hippocampus. Mol. Neurobiol. 55, 164–172. 10.1007/s12035-017-0730-0 28840469 PMC5808855

[B29] Pellerin L. Bergersen L. H. Halestrap A. P. Pierre K. (2005). Cellular and subcellular distribution of monocarboxylate transporters in cultured brain cells and in the adult brain. J. Neurosci. Res. 79, 55–64. 10.1002/jnr.20307 15573400

[B30] Pérez-Escuredo J. van Hée V. F. Sboarina M. Falces J. Payen V. L. Pellerin L.. (2016). Monocarboxylate transporters in the brain and in cancer. Biochim. Biophys. Acta 1863, 2481–2497. 10.1016/j.bbamcr.2016.03.013 26993058 PMC4990061

[B31] Perkins M. Wolf A. B. Chavira B. Shonebarger D. Meckel J. P. Leung L.. (2016). Altered energy metabolism pathways in the posterior cingulate in young adult apolipoprotein E varepsilon4 carriers. J. Alzheimers Dis. 53, 95–106. 10.3233/jad-151205 27128370 PMC4942726

[B32] Ranganathan M. DeMartinis N. Huguenel B. Gaudreault F. Bednar M. M. Shaffer C. L.. (2017). Attenuation of ketamine-induced impairment in verbal learning and memory in healthy volunteers by the AMPA receptor potentiator PF-04958242. Mol. Psychiatry 22, 1633–1640. 10.1038/mp.2017.6 28242871

[B33] Sassano-Higgins S. Baron D. Juarez G. Esmaili N. Gold M. (2016). A review of ketamine abuse and diversion. Depress Anxiety 33, 718–727. 10.1002/da.22536 27328618

[B34] Solís-Maldonado M. Miro M. P. Acuna A. I. Covarrubias-Pinto A. Loaiza A. Mayorga G.. (2018). Altered lactate metabolism in Huntington’s disease is dependent on GLUT3 expression. CNS Neurosci. Ther. 24, 343–352. 10.1111/cns.12837 29582588 PMC6490050

[B35] Sun L. Lam W. P. Wong Y. W. Lam L. H. Tang H. C. Wai M. S.. (2011). Permanent deficits in brain functions caused by long-term ketamine treatment in mice. Hum. Exp. Toxicol. 30, 1287–1296. 10.1177/0960327110388958 21056951

[B36] Sun Y. Yang J. Hu X. Gao X. Li Y. Yu M.. (2018). Lanthanum chloride reduces lactate production in primary culture rat cortical astrocytes and suppresses primary co-culture rat cortical astrocyte-neuron lactate transport. Arch. Toxicol. 92, 1407–1419. 10.1007/s00204-017-2148-x 29264619

[B37] Suzuki A. Stern S. A. Bozdagi O. Huntley G. W. Walker R. H. Magistretti P. J.. (2011). Astrocyte-neuron lactate transport is required for long-term memory formation. Cell 144, 810–823. 10.1016/j.cell.2011.02.018 21376239 PMC3073831

[B38] Tan S. Rudd J. A. Yew D. T. (2011). Gene expression changes in GABA_A_ receptors and cognition following chronic ketamine administration in mice. PLoS One 6:e21328. 10.1371/journal.pone.0021328 21712993 PMC3119682

[B39] Tosta C. L. Silote G. P. Fracalossi M. P. Sartim A. G. Andreatini R. Joca S. R. L.. (2019). S-ketamine reduces marble burying behaviour: involvement of ventromedial orbitofrontal cortex and AMPA receptors. Neuropharmacology 144, 233–243. 10.1016/j.neuropharm.2018.10.039 30385254

[B40] Vannucci S. J. Simpson I. A. (2003). Developmental switch in brain nutrient transporter expression in the rat. Am. J. Physiol. Endocrinol. Metab. 285, E1127–E1134. 10.1152/ajpendo.00187.2003 14534079

[B42] Wang R. R. Jin J. H. Womack A. W. Lyu D. Kokane S. S. Tang N.. (2014). Neonatal ketamine exposure causes impairment of long-term synaptic plasticity in the anterior cingulate cortex of rats. Neuroscience 268, 309–317. 10.1016/j.neuroscience.2014.03.029 24674848 PMC4026048

[B43] Wang W. Liu L. Yang X. Gao H. Tang Q. K. Yin L. Y.. (2019). Ketamine improved depressive-like behaviors *via* hippocampal glucocorticoid receptor in chronic stress induced- susceptible mice. Behav. Brain Res. 364, 75–84. 10.1016/j.bbr.2019.01.057 30753876

[B41] Wang J. Zhou M. Wang X. Yang X. Wang M. Zhang C.. (2014). Impact of ketamine on learning and memory function, neuronal apoptosis and its potential association with miR-214 and PTEN in adolescent rats. PLoS One 9:e99855. 10.1371/journal.pone.0099855 24914689 PMC4051782

[B44] Whitlock J. R. Heynen A. J. Shuler M. G. Bear M. F. (2006). Learning induces long-term potentiation in the hippocampus. Science 313, 1093–1097. 10.1126/science.1128134 16931756

[B45] Xu K. Lipsky R. H. (2015). Repeated ketamine administration alters N-methyl-D-aspartic acid receptor subunit gene expression: implication of genetic vulnerability for ketamine abuse and ketamine psychosis in humans. Exp. Biol. Med. 240, 145–155. 10.1177/1535370214549531 25245072 PMC4469194

[B46] Yeung L. Y. Wai M. S. Fan M. Mak Y. T. Lam W. P. Li Z.. (2010). Hyperphosphorylated tau in the brains of mice and monkeys with long-term administration of ketamine. Toxicol. Lett. 193, 189–193. 10.1016/j.toxlet.2010.01.008 20093173

[B47] Zhang Y. Xue Y. Meng S. Luo Y. Liang J. Li J.. (2016). Inhibition of lactate transport erases drug memory and prevents drug relapse. Biol. Psychiatry 79, 928–939. 10.1016/j.biopsych.2015.07.007 26293178

[B48] Zheng W. Zhou Y. L. Liu W. J. Wang C. Y. Zhan Y. N. Li H. Q.. (2019). Neurocognitive performance and repeated-dose intravenous ketamine in major depressive disorder. J. Affect. Disord. 246, 241–247. 10.1016/j.jad.2018.12.005 30590286

