# Supplementary material for: Redistribution of Monocarboxylate 1 and 4 in Hippocampus and Spatial Memory Impairment Induced by Long-term Ketamine Administration
Source: Front Behav Neurosci. 2020 Apr 17;14:60. doi: 10.3389/fnbeh.2020.00060 (PMC7181955; doi:10.3389/fnbeh.2020.00060)
Supplement: Supplementary file 1 [file Table_1.docx]

Table S1. Details of daily ketamine or saline injections of mice.

| **group** | **saline** | **ket1** | **ket2** |
| --- | --- | --- | --- |
| **dilution** | natural saline solution without dilution | ketamine hydrochloride 5 times diluted by natural saline | ketamine hydrochloride 2.5 times diluted by natural saline |
| **Volume**  **(μL)** | mouse body weight （g）*3 | mouse body weight （g）*3 | mouse body weight （g）*3 |

The volume of ketamine hydrochloride was 100mg/ 2ml in each ampoule bottle. Body weight of mice were measured every 3 days. Solutions of ketamine or saline were prepared by micropipettor before intraperitoneal injections of mice every day. The volume of injections was adjusted by the “body weight index”. In this experiment, the “body weight index” was 3, which means the volume of the final injections was 3 times of the body weight. All the efforts were made to minimize the inaccuracy and suffering.
