# Supplementary material for: Redistribution of Monocarboxylate 1 and 4 in Hippocampus and Spatial Memory Impairment Induced by Long-term Ketamine Administration
Source: Front Behav Neurosci. 2020 Apr 17;14:60. doi: 10.3389/fnbeh.2020.00060 (PMC7181955; doi:10.3389/fnbeh.2020.00060)
Supplement: Supplementary file 2 [file Table_2.docx]

| **Gene name** | **Primer sequences** |
| --- | --- |
| *MCT1* | Forward: 5′-AGAAGCCAAAGGAGACGATG-3′ |
|  | Reverse: 5′-GATGGTTTTGGATGTCGTGG-3′ |
| *MCT2* | Forward: 5′-ATCAATCGCTGTGCTCTCG-3′ |
|  | Reverse: 5′- GCCACTTTGTGTTGACGTG-3′ |
| *MCT4* | Forward: 5′-TGAGAGCACTTAAAGTCGCC-3′ |
|  | Reverse: 5′-GTATTCAGTCCAGCCTACTCG-3′ |
| *GAPDH* | Forward: 5′-GGCACAGTCAAGGCTGAGAATG-3′ |
|  | Reverse: 5′-ATGGTGGTGAAGACGCCAGTA-3′ |

**Table S2.** details of MCT1, MCT2, MCT4 and GAPDH primer sequences.
