# Supplementary material for: Redistribution of Monocarboxylate 1 and 4 in Hippocampus and Spatial Memory Impairment Induced by Long-term Ketamine Administration
Source: Front Behav Neurosci. 2020 Apr 17;14:60. doi: 10.3389/fnbeh.2020.00060 (PMC7181955; doi:10.3389/fnbeh.2020.00060)
Supplement: Supplementary file 4 [file Data_Sheet_1.docx]

**Figure 1. Schedule of ketamine administration and behavioral assessment**. Ketamine daily administration for 180 days (6 months). Morris Water Maze (MWM) test began on Day 181 and lasted until Day 188 of the study, Radial Arm Maze (RAM) test started on Day 181 and lasted until on Day 183. We surveyed the sessions of escape latency (Day 183–187) and probe trials (Day 188) of mice to assess for spatial memory performance in MWM test. Meanwhile, total errors and time spent to finish a session (Day 92–93 and Day 182–183) of mice in the RAM test were conducted to assess for spatial working memory performance. Animals were euthanized on Day 184 and 189, and brain tissue was collected in the −80°C freezer.

**Figure 2. Spatial memory performance of mice in MWM test following six months of ketamine administration with different doses of ketamine 30 mg/kg and 60 mg/kg.** (A) Significant increase of latency time to reach the target in Morris Water Maze test invisible platform trails in ket2 group in the training day 2, 3, 4, 5 after six months of administration paradigm (**p* < 0.01, ***p* < 0.01) and ket1group (30 mg/kg) saw increase of latency time to reach the target only in the training day 3 (^#^*p* < 0.01). (B) Significant decline of the times of crossing the former platform location was observed in ket2 group comparing with control group (***p* < 0.01). (C-E) Representative swimming paths by mice with different treatments in probe trial tests.

**Figure 3. Spatial working memory performance of mice in RAM tests following six months of ketamine administration with different doses of ketamine 30 mg/kg and 60 mg/kg.** (A) No significance of time spent in finishing a session were seen in three groups. (B) Significant increase of total errors in one session to accomplish a Radial Arm Maze test in ket2 group comparing with control group (**p* < 0.05). (C-E) Representative searching tracks of mice with different treatments in the Radial Arm Maze tests acquisition trails.

**Figure 4. Changes of hippocampal membrane MCT1, MCT4 and MCT2 protein levels after six months of ketamine administration in different groups as reveled by Western blot.** (A, B) Significant decline of MCT1 expression levels were seen in ket2 group comparing with ket1 group and control group respectively (^##^*p* < 0.01, ***p* < 0.01). (C, D) Significant decline of MCT4 expression levels were seen in ket2 group comparing with control group and ket1group respectively (**p* < 0.05, ^##^*p* < 0.01). obviously increase of MCT4 expression levels were seen in ket1 group comparing with control group (**p* < 0.05). (E, F) Significant increase of MCT2 expression levels were seen in ket2 and ket1 group comparing with control group respectively (***p* < 0.01, **p* < 0.05).

**Figure 5. Changes of hippocampal cytoplasm MCT1, MCT4 and MCT2 protein levels after six months of ketamine administration in different groups as reveled by Western blot.** (A, B) Significant increase of MCT1 expression levels were seen in ket2 and ket1group comparing with control group respectively (***p* < 0.01). (C, D) Significant increase of MCT4 expression levels were seen in ket2 and ket1 group comparing with control group (**p* < 0.05, ***p* < 0.01). (E, F) Significant increase of MCT2 expression levels were seen in ket2 and ket1 group comparing with control group respectively (***p* < 0.01).

**Figure 6. Changes of hippocampal MCT1, MCT4 and MCT2 mRNA levels after six months of ketamine administration in different groups.** (A) No significance of MCT1 mRNA expression levels were seen in three groups. (B) Significant increase of MCT2 mRNA expression levels were seen in ket2 and ket1 group comparing with control group (**p* < 0.05, ***p* < 0.01). (C) No significance of MCT4 mRNA expression levels were seen in three groups.

**sFigure 1. Statistics of body weight after 6-month injection paradigm.** No significance of body weight of mice was seen in three groups after 6 months of ketamine or saline intraperitoneal injections.
